# Air seal performance of personalized and statistically shaped 3D-printed face masks compared with market-available surgical and FFP2 masks

**DOI:** 10.1038/s41598-021-98963-0

**Published:** 2021-09-29

**Authors:** Julian Nold, Marc C. Metzger, Steffen Schwarz, Christian Wesemann, Gregor Wemken, Stefano Pieralli, Florian Kernen, Julia Weingart, Carl G. Schirmeister, Stefan Schumann, Stefan Schlager, Benedikt C. Spies

**Affiliations:** 1 Department of Prosthetic Dentistry, Center for Dental Medicine, Faculty of Medicine, Medical Center, University of Freiburg, Hugstetter Str. 55, 79106 Freiburg, Germany; 2 Department of Oral and Maxillofacial Surgery, Center for Dental Medicine, Faculty of Medicine, Medical Center, University of Freiburg, Hugstetter Str. 55, 79106 Freiburg, Germany; 3 Institute for Macromolecular Chemistry, Freiburg Materials Research Center, University of Freiburg, Stefan-Meier-Str. 21, 79104 Freiburg, Germany; 4 Basell Sales & Marketing B.V., LyondellBasell Industries, Industriepark Höchst, 65926 Frankfurt am Main, Germany; 5 Department of Anesthesiology and Critical Care, Faculty of Medicine, Medical Center, University of Freiburg, Hugstetter Str. 55, 79106 Freiburg, Germany; 6 Department of Biological Anthropology, Medical Center, University of Freiburg, Hebelstraße 29, 79106 Freiburg, Germany

**Keywords:** Health care, Medical research

## Abstract

The ongoing COVID-19 pandemic has revealed alarming shortages of personal protective equipment for frontline healthcare professionals and the general public. Therefore, a 3D-printable mask frame was developed, and its air seal performance was evaluated and compared. Personalized masks (PM) based on individual face scans (n = 8) and a statistically shaped mask (SSM) based on a standardized facial soft tissue shape computed from 190 face scans were designed. Subsequently, the masks were additively manufactured, and in a second step, the PM and SSM were compared to surgical masks (SM) and FFP2 masks (FFP2) in terms of air seal performance. 3D-printed face models allowed for air leakage evaluation by measuring the pressure inside the mask in sealed and unsealed conditions during a breathing simulation. The PM demonstrated the lowest leak flow (p < 0.01) of inspired or expired unfiltered air of approximately 10.4 ± 16.4%, whereas the SM showed the highest (p < 0.01) leakage with 84.9 ± 7.7%. The FFP2 and SSM had similar values of 34.9 ± 18.5% leakage (p > 0.68). The developed framework allows for the time- and resource-efficient, on-demand, and in-house production of masks. For the best seal performance, an individually personalized mask design might be recommended.

## Introduction

The ongoing COVID-19 pandemic continues to spread around the world, and healthcare systems still face an associated increased need for personal protective equipment (PPE). Both healthcare professionals and the general public are faced with a critical shortage of PPE, particularly face masks^1,2^. This has resulted in a call for an Emergency Use Authorization (EUA) in the US (FD&C Act)^3^ and the EU^4^.

Standard surgical masks (SM) are designed to prevent the wearer from transmitting droplets to others and into the surgical field. Their filterability and sealing properties are low^5–8^. SMs are secured by either ear loops or with ties and mainly consist of melt-blown fabric. Most feature a metal insert that can be shaped to improve the fit around the nose. While FFP2/N95 respirators also use melt-blown fabric and inserts to improve the fit around the nose, their elastic straps result in a tighter face seal and therefore an improved fit and filtering performance^9^.

For frontline healthcare professionals working with COVID-19 patients, face masks with a high filtration capacity and a leakage-proof fit are needed. The protection level of respirators is assessed by evaluating the filtration efficiency, face seal leakage and fit factors as well as additional national standards defined by the ASTM F2100^10^ (USA) and DIN EN 14683:2019^11^ (EU) for surgical masks and the NIOSH 42 CFR 84^12^ (USA) and EN 149:2001^13^ (EU) for N95/FFP2 masks. Only N95 respirators (corresponding to the FFP2 standard) and N99 respirators (corresponding to the FFP3 standard) meet the standards required for protection against aerosol and droplet transmission^14^.

The air seal performance of standard masks has already been evaluated and compared in various studies, showing the importance of a proper fit^15,16^. A universal one-piece facemask with a perfect fit seems improbable. This has been confirmed in in vivo investigations examining the fit of commercially available facemasks^8,17^. However, further testing is needed to evaluate the seal performance of standard masks versus new mask shape designs.

3D printing has been widely applied in healthcare and medical research, spanning from bioprinting bone scaffolds for enhanced bone regeneration^18^ to printing medical models for surgeons^19^. It offers the unique possibility of a cost-effective and individual manufacturing method for specific applications^20^.

The aim of this study was to develop a workflow to be shared with the community to design and manufacture personalized 3D-printed masks based on individual face scans. Second, a mask design was created that allowed for large-scale production based on 190 face scans by means of statistical shape modeling. Third, the seal performance of the developed mask designs was compared with that of commonly available surgical masks and FFP2 respirators. Fourth, we evaluated the feasibility of the rapid 3D printing of face mask frameworks. The null hypothesis assumed that none of the masks used in the study would show differences in seal performance.

## Materials and methods

### Computer-aided design and computer-aided manufacturing of test faces

**Figure 1:**
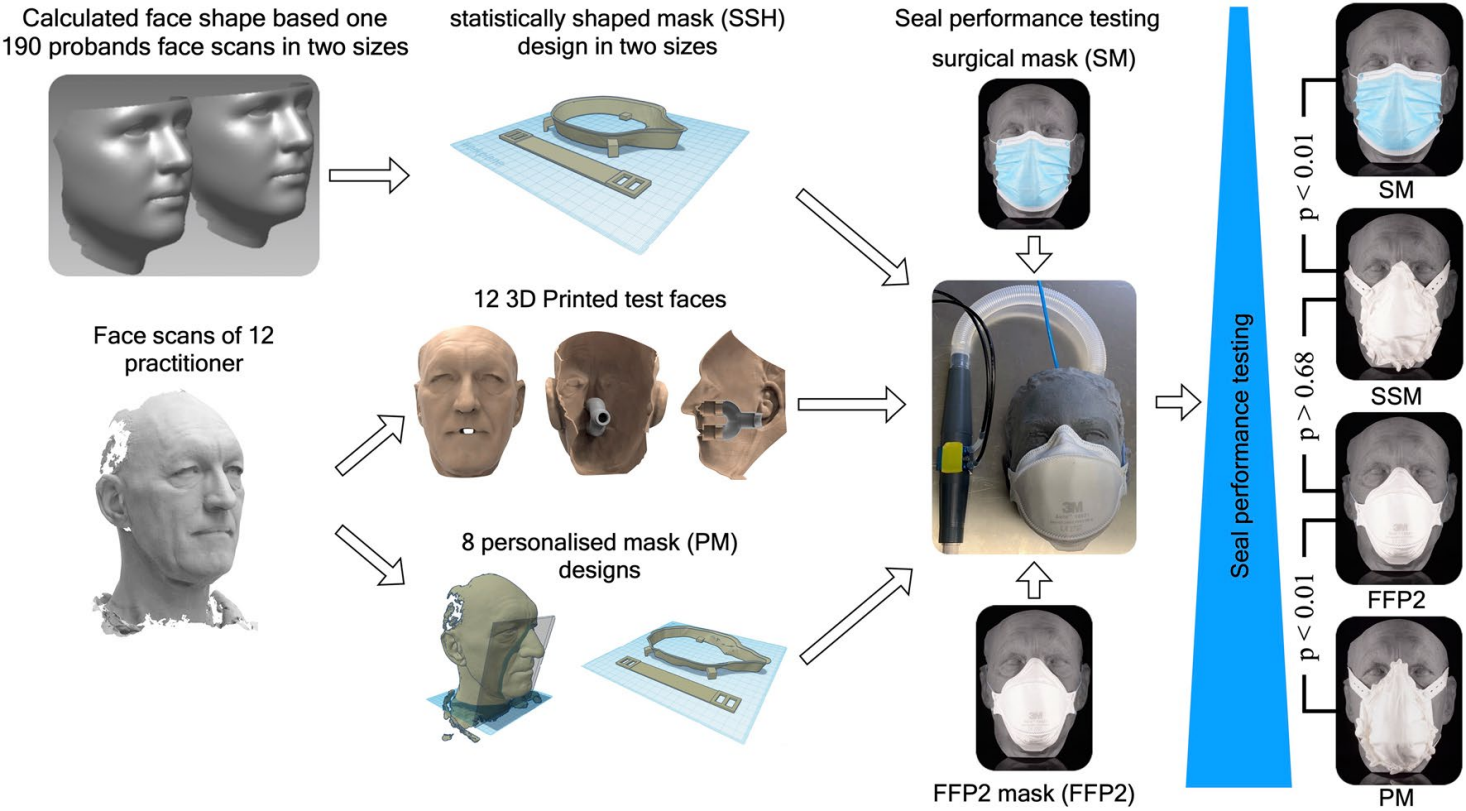
Graphical Abstract summarizing the workflow of mask and test face design, seal performance testing and the archived results.

**Figure 2:**
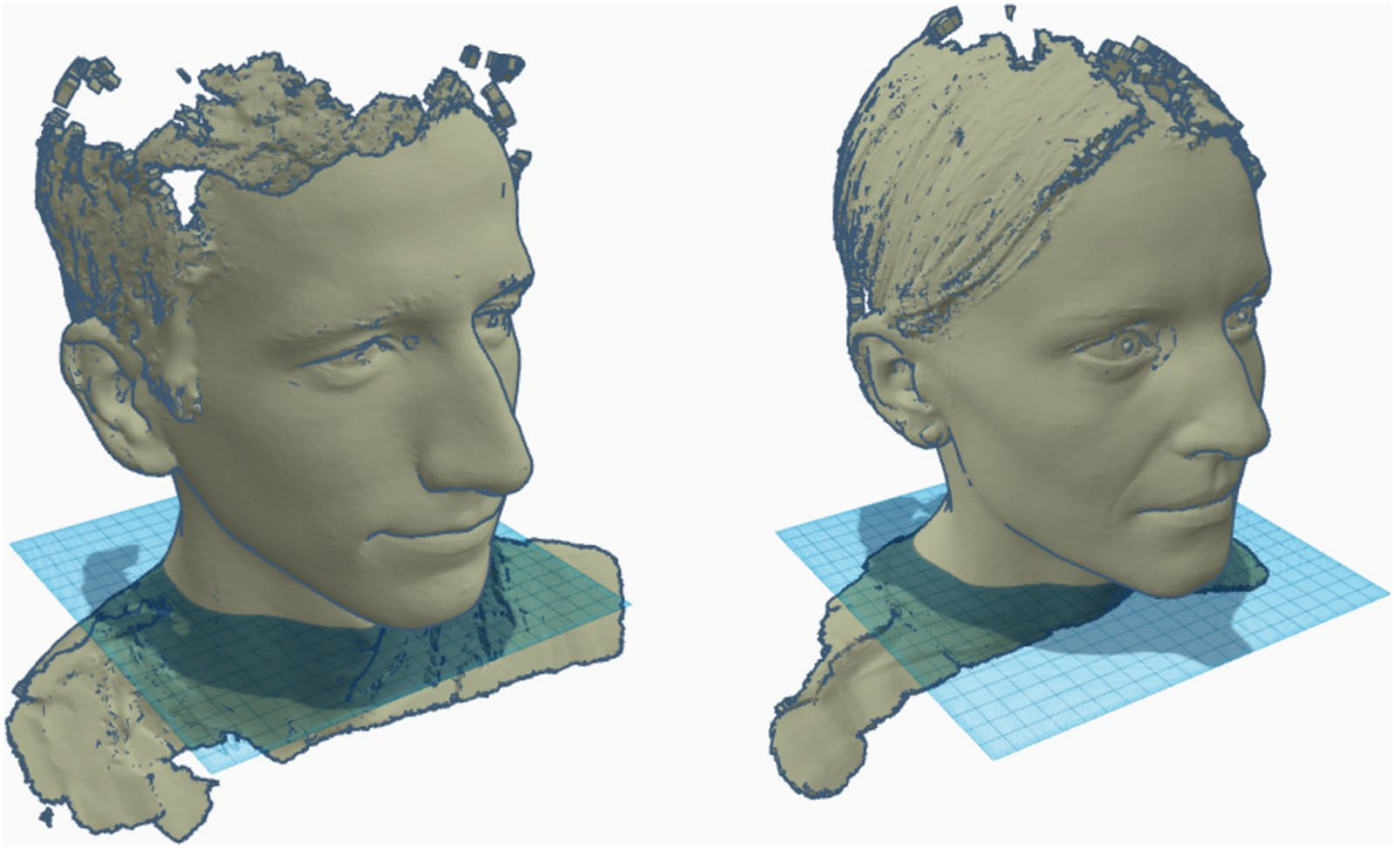
Two of the 12 face scans used to create seal performance test faces.

To allow for comparison (Fig. 1) with individually designed masks, the faces of 12 employees were digitalized (FaceHunter, Zirkonzahn, South Tyrol, Italy), representing a wide variety of face shapes (Fig. 2).

**Figure 3:**
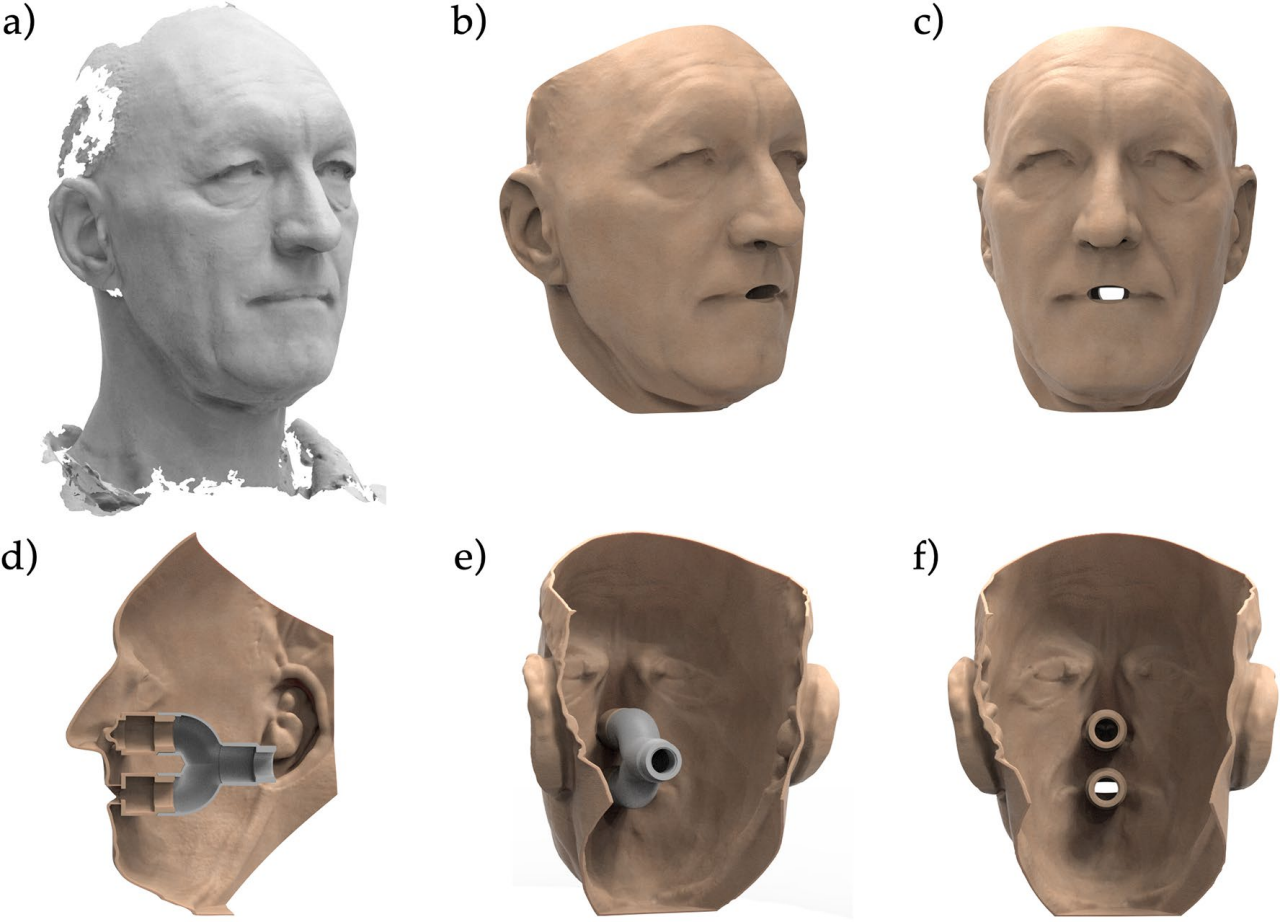
Practitioner’s face scan **(a)** and one test face design with added holes in the nose and mouth **(b,c)**. A tube system and an adaptor were designed and printed for connection to the pump **(d–f)**.

In cooperation with 3D-LABS (St. Georgen, Baden-Württemberg, Germany), the surface files of the digitized faces were loaded into 3D modeling software (Geomagic Freeform, 3D Systems, Rock Hill, South Carolina, United States) to create the test faces for seal performance testing. This included adding an inward offset of 2 mm to create a rigid and printable design while retaining the dimensional accuracy of the original surface. Additionally, perforations in the nose (diameter: 7 mm) and mouth (width: 22 mm, height: 7 mm) were integrated. Tubes with an inner diameter of 7 mm for the nose holes and a tube with an inner diameter of 14 mm for the mouth with connectors at the inward end were incorporated. Furthermore, an adapter was designed to join the nose and the mouth tube connections to a single connection. This allowed for easy access of all tubes, which was needed for insertion of the pressure sensor tube (polyurethane tube, 4 × 2.5 mm, Sang-A Pneumatic Co., Daegu, Korea) through one side of the nose while retaining the ability to use both the nose and mouth for airflow testing. For the realistic fit evaluation of earloop-worn surgical masks, the area behind the ear was carved out to allow for a reliable retention spot (Fig. 3). After completing the test face designs, all 12 face models were additively manufactured using a MultiJet Printer (HP 4200, HP, Palo Alto, California, U.S.).

### Statistical shape models for estimating the midfacial shape

For surface registration, statistical analysis and generation of the respective surface shapes, the mathematical/statistical platform R (R Core Team, 2020, Vienna, Austria) and the Rpackages Morpho, Rvcg (Schlager, 2017, Germany), mesheR (Schlager, 2015a, Germany) and RvtkStatismo (Schlager, 2015b, Germany) were used. To determine an average midfacial shape, a sample size consisting of 190 3D-surface scans of European adult faces (Artec MHT portable, Artec, Luxembourg) was used to build sex-specific shape models. The data consisted of 147 females (range: 16–70 years; mean 34.2 ± 15.2 years, average BMI 23.1) and 43 males (range: 15–76 years; mean 35.3 ± 14.3 years, average BMI 24.6).

**Figure 4:**
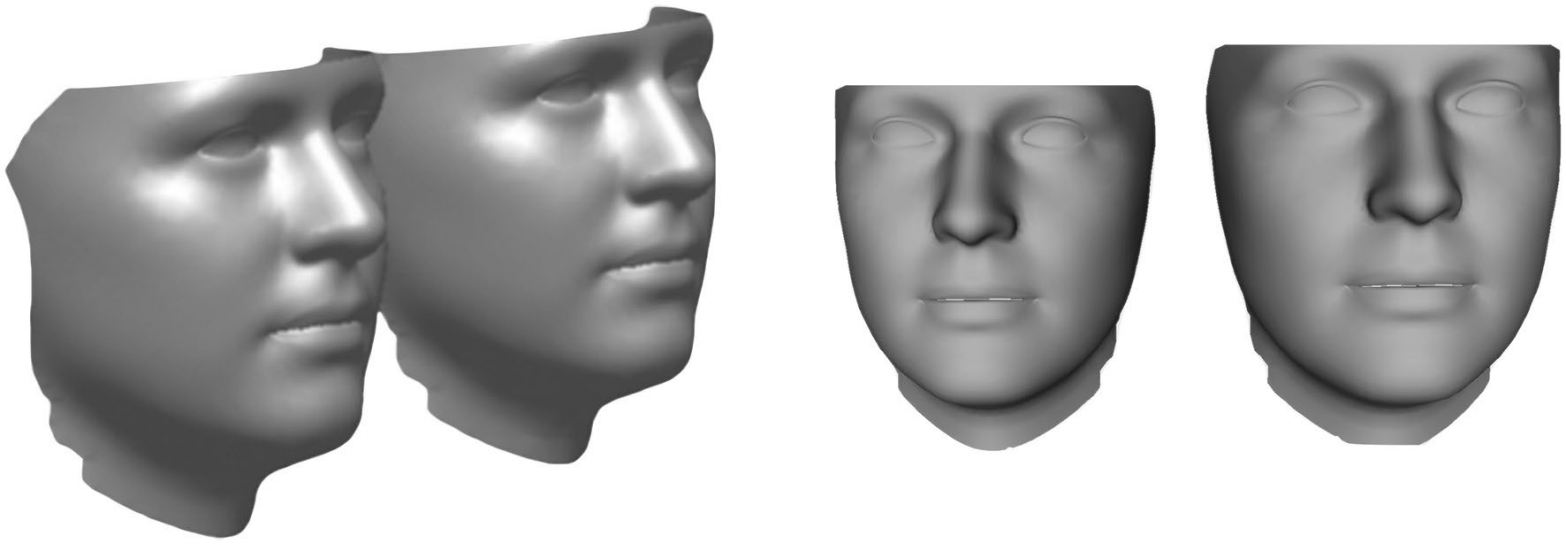
Sex-neutral face shape models in two sizes (small and large) calculated using the R Software (R Core Team, R: A Language and Environment for Statistical Computing, R Foundation for Statistical Computing, Vienna, Austria, 2020, https://www.R-project.org).

After a Procrustes alignment based on the registered meshes’ vertices, the average shape was computed for both sexes. The averaging of these estimations yielded a sex-neutral face. For scaling to the appropriate size, the sex-neutral shape was then scaled to the average centroid size of each sex, resulting in two sizes, small and large. The calculated facial surfaces were imported into Rapidform software (Inus Technology, Seoul, South Korea) (Fig. 4), and a forward offset of 1 mm was generated to create a volume out of the surface, which was finally saved as an STL file.

### Computer-aided design of the statistically shaped mask

**Figure 5:**
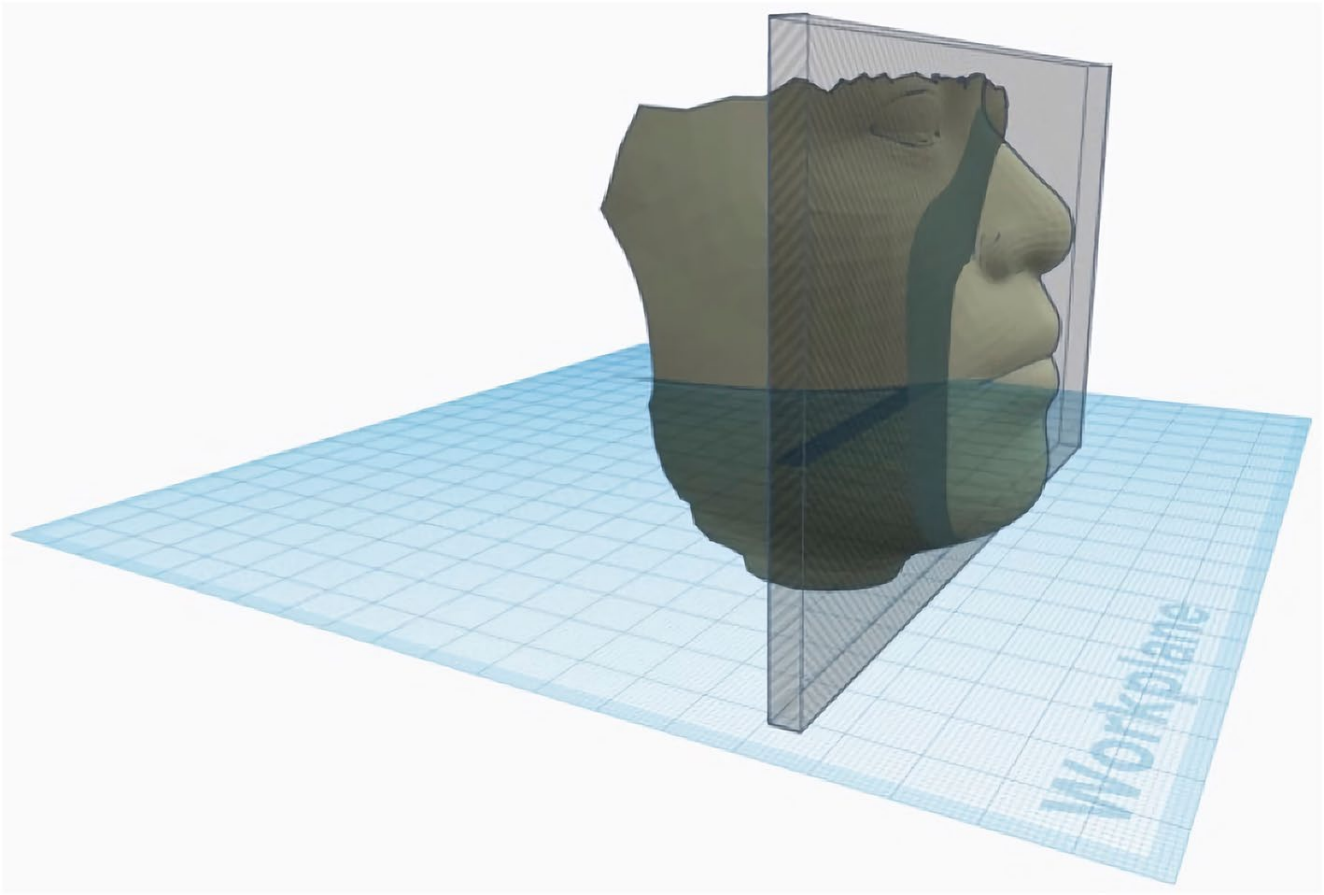
An 8 mm slice ranging from the glabella to the menton is cropped to serve as a precursor for the statistically shaped mask based on the calculated average face.

For the computer-aided design (CAD) of the statistically shaped mask (SSM) frame, the STL data of the reference faces were imported into browser-based 3D CAD software (Tinkercad, Autodesk, California, USA) and aligned to the horizontal plane. A two-piece mask design was chosen, combining a mask frame with a horizontal tensioner that allows for easy mounting of the filter cloth. To create the frame part of the mask, an 8 mm thick vertical slice ranging from the glabella to the menton was cropped from the volume of the reference faces and served as a precursor (Fig. 5).

**Figure 6:**
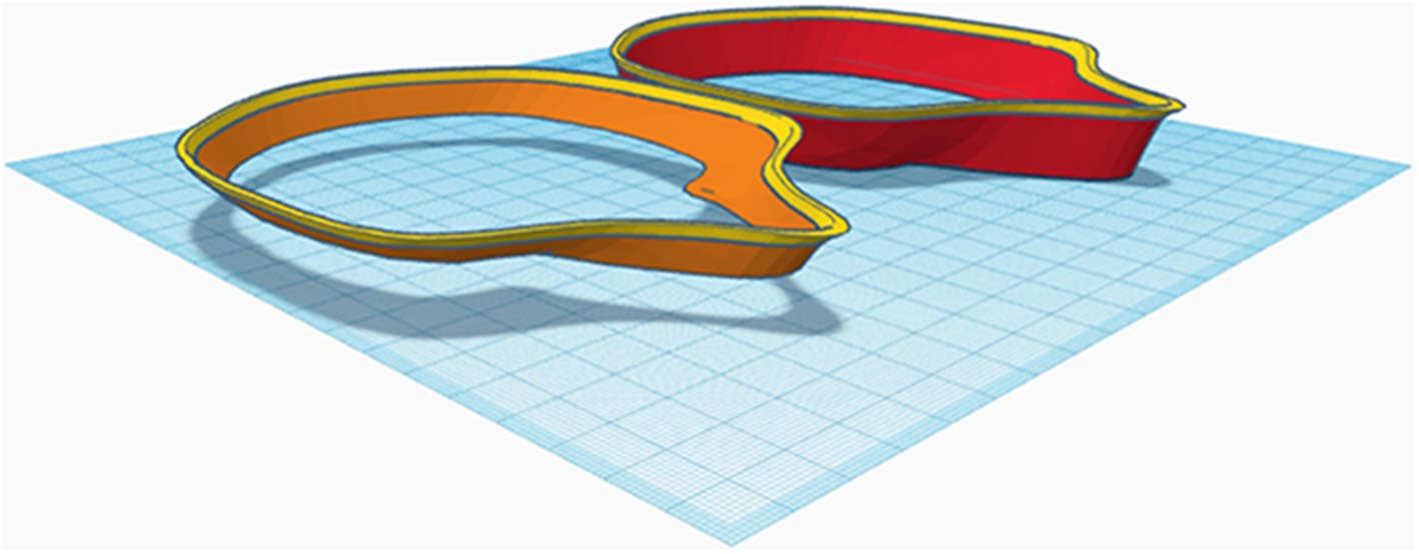
For demonstration purposes, the original slice has been cut into a 2 mm proximal (yellow) and a 6 mm ventral (orange) part; the ventral part has been stretched ventrally (red) and aligned with the unstretched proximal part (yellow) prior to grouping.

On the resulting 8 mm slice, the proximal part (6 mm) was cut, ventrally stretched (13 mm), and finally merged with the lateral part of the original slice (2 mm) (Fig. 6).

**Figure 7:**
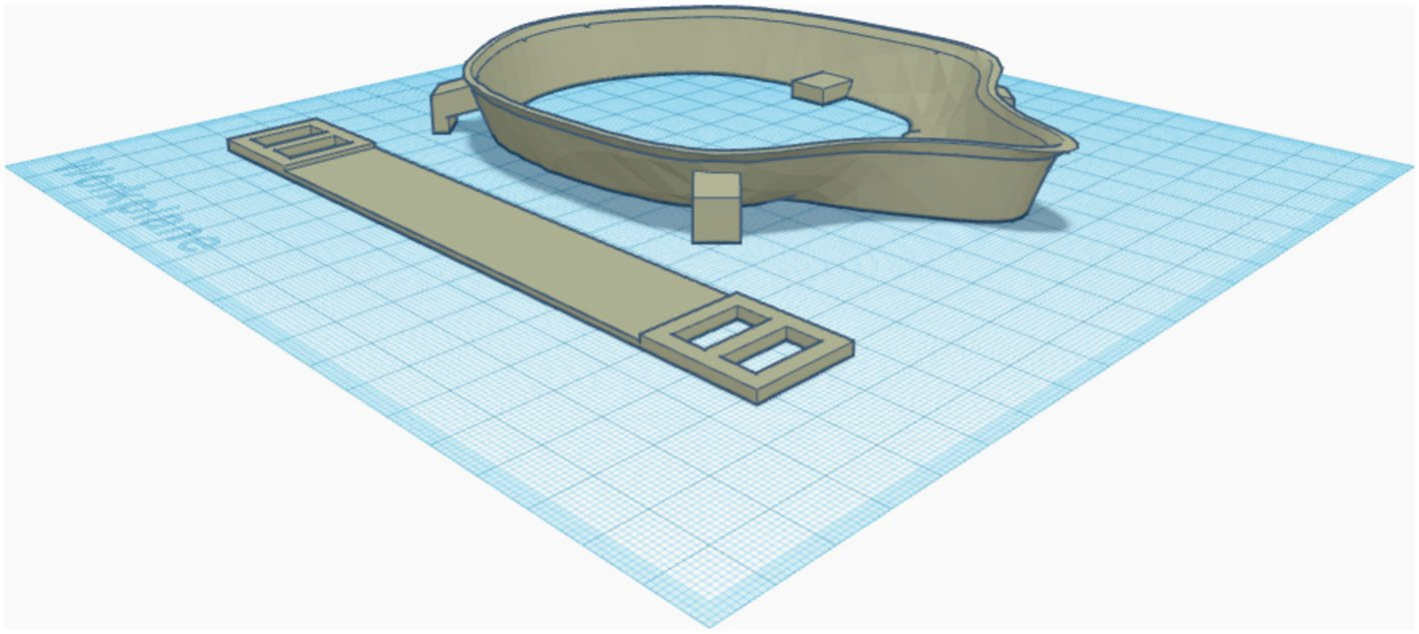
Finished 3D designs of the statistically shaped mask frame and tensioner.

Rectangles (9 × 11 × 3.5 mm) were attached to the proximal side of the frame at the level of the labial angle for retention of the horizontal tensioner. To attach the filter cloth (Freudenberg, Weinheim, Germany) and buttonhole elastics (12 mm; Prym, Stolberg, Germany), 4 attachments consisting of grouped rectangles (3 × 8 × 10 mm and 3 × 7 × 10 mm) were connected to the outer surface of the frame. For the CAD of the tensioner, a rectangle (150 × 20 × 1.5 mm) was built and reinforced by another rectangle measuring 0.5 mm in height and 23 mm in length at both ends. Two rectangular holes (8 × 15 mm) attached to the referring retention on the frame were cut into the reinforced area. Finally, the data were exported as a STL file and imported to open-source slicing software (Cura 4.5, Ultimaker). For improved bed adhesion, a brim of 8 outlines was used, and the GCODE was generated for 3D printing (Fig. 7).

### Design of personalized face masks based on individual face scans

Eight of the twelve scans used for creating the models used for measurement were selected for designing personalized face masks (PM). Eight personalized mask frames were designed following the same approach used for creating the statistically shaped masks. The same tensioner design was used with the personalized masks.

### Manufacturing and assembly of 3D-printed masks

For 3D printing of the mask frames, a polypropylene (PP) copolymer (*Purell* EP274P, LyondellBasell, Rotterdam, Netherlands, MFR = 15 g (10 min)^−1^, T_m_ = 165 °C, Vicat softening temperature (VST A50) = 142 °C, Young’s modulus (3D printed) = 1000 MPa) was used, allowing a wide temperature range for the application. Due to the certified biocompatibility (ISO 10993 & class VI according to USP 88) and the requirements on heavy metal content and additives in close collaboration with pharmaceutical associations, irritation in case of direct contact of the PP with skin or wounds was widely excluded (Ph. Eur. 3.1.3 & 3.1.6, USP 661.1).

**Figure 8:**
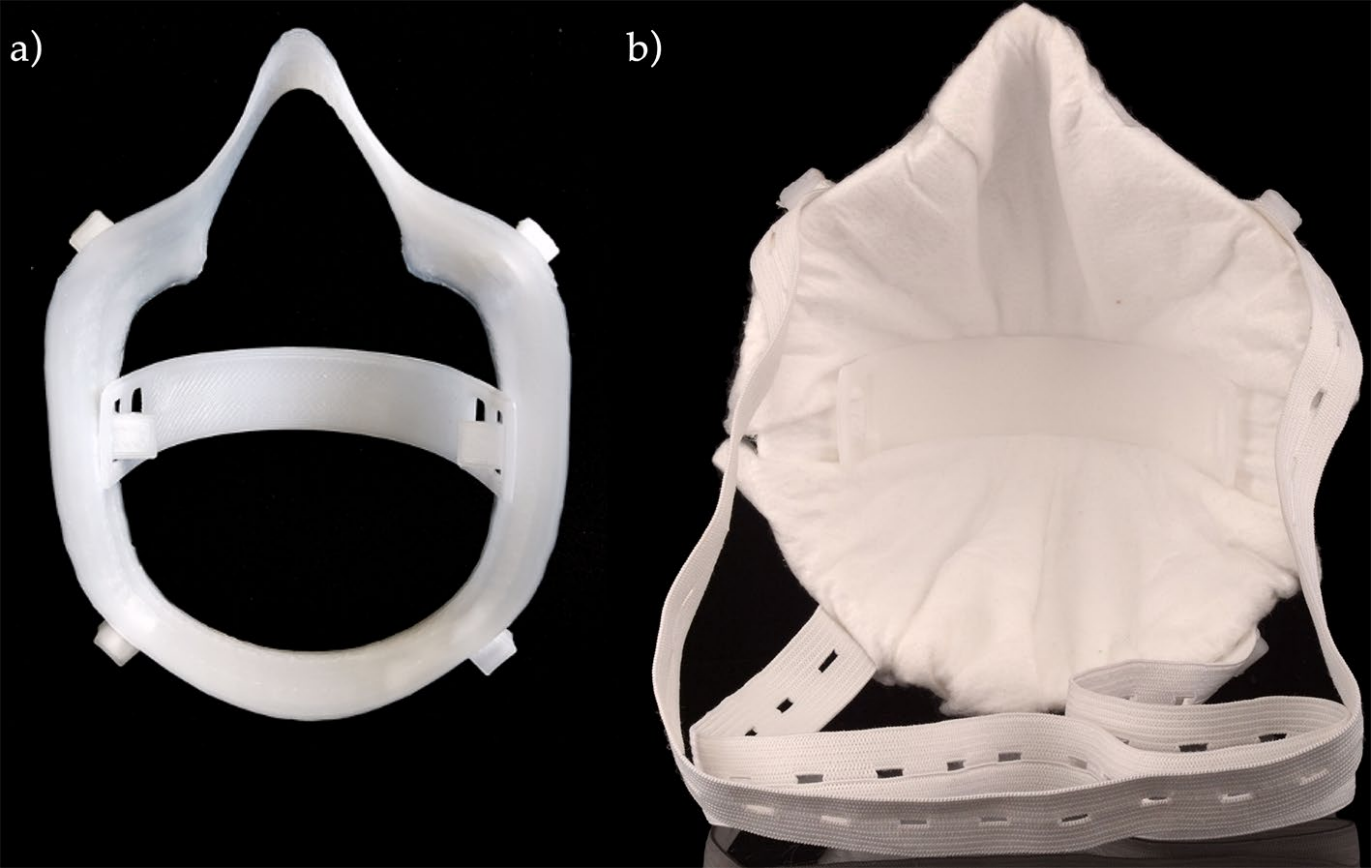
**(a)** Test fitting of the 3D-printed statistically shaped mask frame and tensioner; **(b)** fully assembled statistically shaped mask incorporating a sample filter cloth.

A fused filament fabrication (FFF) printer (Ultimaker S5, Ultimaker B.V., Netherlands) was equipped with a steel nozzle (0.8 mm diameter) and fiber-reinforced PP adhesive tape (Scotch extreme packaging tape, 3 M, USA) as the print bed. Printing was performed at a nozzle temperature of 210 °C, build plate temperature of 35 °C, printing speed of 35 mm/s, wall thickness of 1.5 mm, layer height of 0.2 mm, and 100% infill. The application of support structures was not necessary. Up to four mask frames were printed simultaneously. After printing, the brim was removed from the frame. The horizontal retainer was prebent to an end-to-end distance of 80 mm (Fig. 8).

A cutting pattern was designed for each framework size, including the hole position needed for filter retention. After fixing the melt-blown filter cloth with its holes over the attachment feet and adding the tensioner, rubber bands were added, additionally securing the filter cloth.

### Seal performance evaluation and comparison with surgical and FFP2 masks

**Figure 9:**
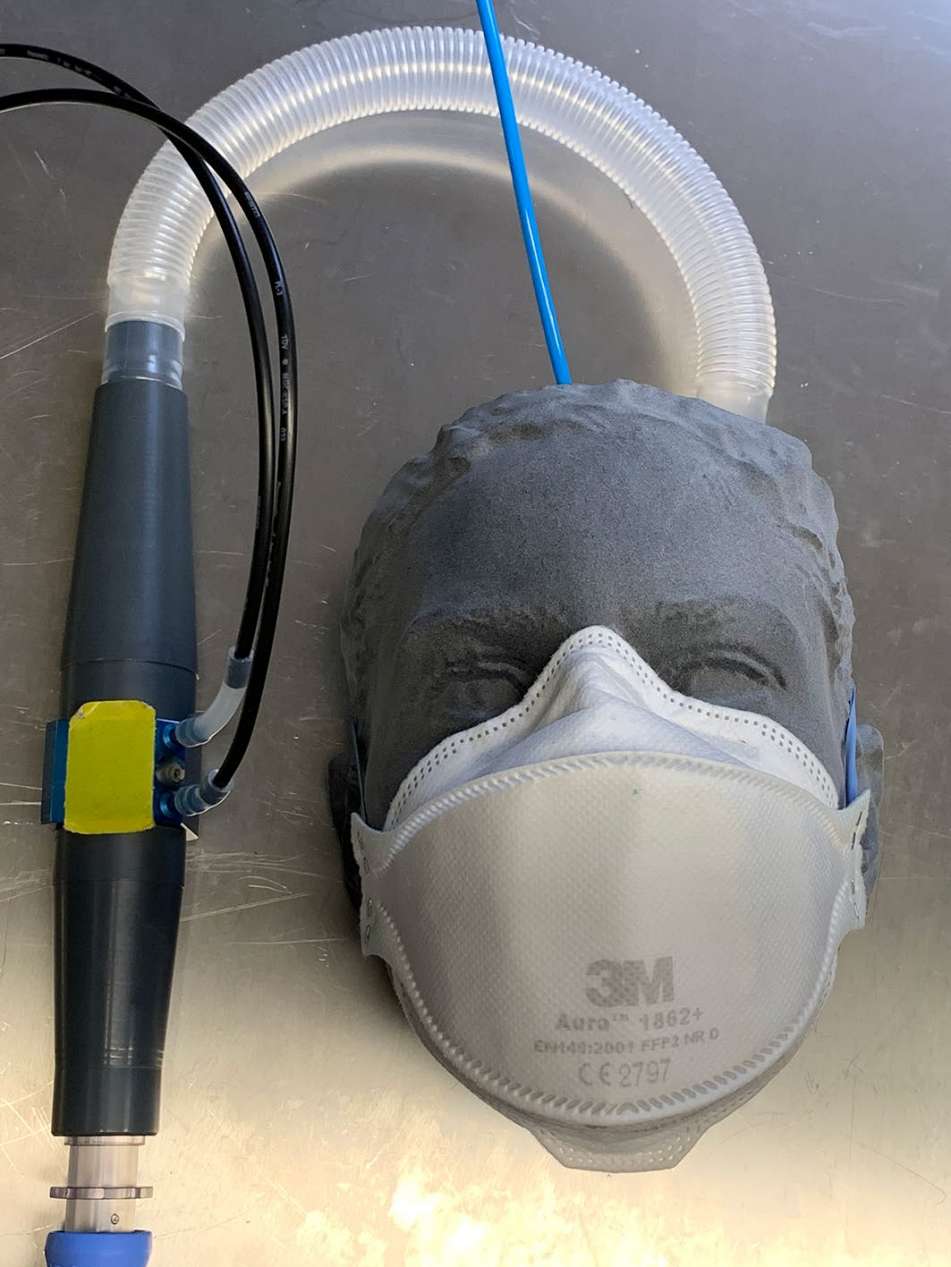
Measurement setup for the FFP2 mask while simulating human-like breathing. The pressure tubes exiting the obstruction flow meter (black) and the tube coming from the inside of the mask (blue) are connected to a pressure sensor array.

To measure the amount of leaked air, a known breathing-like airflow cycle was generated with a sine wave-like motion at 0.33 Hz and a stroke volume of 1500 ml. This was achieved via a purpose-built, computer-controlled linear motor actuated piston pump. The flow rate was measured via a Fleisch pneumotachograph (Type 2, Dr. Feyves & Gut, Hechingen, Germany), and the pressure was measured using a piezoresistive pressure sensor (Type 2, SI special instruments, Nördlingen, Germany). The sensors were calibrated on a daily basis using a portable calibrator (OM-DM 921, Onneken Mess- und Prüftechnik, Friedrichsdorf, Germany). The in-mask pressure was measured using a tube routed through the back of the 3D-printed test face and into the nose (Fig. 9).

**Figure 10:**
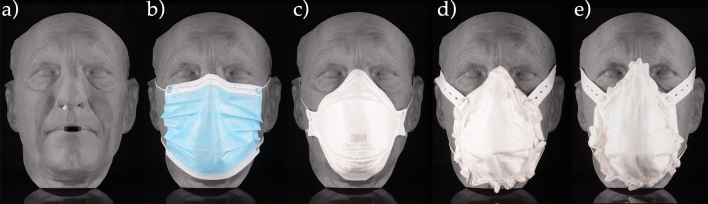
One of the twelve 3D-printed tests faces **(a)** with the air pressure sensor tube installed and **(b)** a surgical mask, **(c)** an FFP2 mask, **(d)** a statistically shaped mask, and **(e)** a personalized mask.

The SSM and the PM were compared to surgical masks (SM, 3 M, Saint Paul, Minnesota, USA) and FFP2 respirator (FFP2, 3 M). Both types of masks are commercially available and in widespread use (Fig. 10).

In addition to the measurements obtained by placing the different masks on the printed faces, as shown in Fig. 10, perfect seal conditions were measured by using a clay like material (Erkogum, Erkodent, Pfalzgrafenweiler, Germany) to eliminate leak flow past the mask, and the resulting pressure readings were recorded while running the same conditions used for the nonsealed tests. The pressure-flow relationships in the perfect seal measurements were used to determine the fluid mechanical conductance of the respective mask within the flow range of ± 800 ml/s. Assuming a simple model of two conductances in parallel, one representing the conductance of the mask filter cloth and one representing the conductance of the air leakage around the mask, we determined the percentage of leak flow from the pressure-flow relationship determined in the nonsealed measurements.

### Statistical analysis

The calculated leakages of the different mask types were analyzed by one-way ANOVA followed by Fisher’s PLSD as a post hoc test. The statistical analyses were performed using a statistical computer program (StatView 5.0, SAS Institute Inc. Cary, NC). The significance level was set to α < 0.05.

### Institutional review board statement

The study was conducted according to the guidelines of the Declaration of Helsinki and approved by the Institutional Ethics Committee of Albert-Ludwig-University Freiburg (264/20, 23.06.2020).

### Informed consent statement

Informed consent was obtained from all subjects involved in the study. Written consent was obtained for publication of the patient's picture shown in Fig. 12.

## Results

### Average face based on sex-neutral shape models

The statistical shape modeling calculation resulted in a sex-neutral form with two sizes, S (height: 205 mm; width: 155 mm) and L (height: 216 mm; width: 163 mm).

### Seal performance of different face masks

The PM revealed the best seal performance, with a leak flow of inspired or expired unfiltered air of approximately 10.4 ± 16.4% (p < 0.01). No differences in sealing performance between the SSM and FFP2 could be calculated (p > 0.68; Table 1). The SM showed the statistically highest amount of leakage (p < 0.01). Mask-related leakage differences were comparable for inspiration and expiration.

**Table 1:** Leakage during inspiration and expiration given for each mask.

Type	N	Inspiration		Expiration	
		Mean (%)	SD (%)	Mean (%)	SD (%)
SM	12	86.074^A^	4.636	83.787^A^	10.004
FFP2	12	34.534^B^	15.948	34.979^B^	16.872
SSMS	7	31.239^B^	19.184	35.810^B^	18.872
SSML	5	34.749^B^	14.799	39.102^B^	13.257
PM	8	7.853^C^	16.737	12.934^C^	16.773

Groups with the same superscript letters (A, B, C) did not differ significantly.

*SD* standard deviation, *SM* surgical mask, *SSMS* statistically shaped mask S, *SSML* statistically shaped mask L, *PM* personalized mask.

### Mask design and 3D printability

The components of the mask were designed following a material- and time-efficient 3D printing process. It allowed for rapidly printing one mask and its retainer in under 60 min using the following printer settings: for PP: 0.8 mm nozzle, 0.2 mm layer height, 35 mm/s; for PLA: 0.4 mm nozzle, 0.3 mm layer height, 50 mm/s. The design is universally combinable with various 3D printing materials. Using PLA filaments allowed for support-free printing without reducing the success rate or print quality. For printing using PP filament, 5% of the used material was needed for improved bed adhesion in the form of a 4 mm brim surrounding the first layer of the print.

## Discussion

In this study, personalized masks based on the individual face scans of 12 practitioners were created. Additionally, an average face contour was calculated using statistical shape modeling with 190 proband face scans. On these bases, a 3D printable statistically shaped mask was developed. To compare fitting properties, test faces were designed on the basis of the 12 practitioner face scans and 3D printed. The 3D printable mask designs were compared in terms of air seal performance with surgical mask and FFP2 respirator. The investigated masks and respirators showed significant differences in terms of air seal performance. Therefore, the null hypothesis had to be rejected. The personalized masks showed significantly better seal performance than all tested masks and respirators. The statistically shaped masks reached a seal performance comparable to that of the FFP2 respirator, while the surgical mask showed the lowest seal performance. The calculation of the statistical shape models of the midface was performed with the intention of overcoming the disadvantages of commercially available masks, which are often characterized by inadequate fitting, a certain degree of leakage and inconvenience. A uniform product design, which is predicted to fit onto 95% (Gaussian distribution) of the analyzed shapes, can be acquired by using statistical shape modeling. In medical image analysis, statistical shape modeling is used as a tool for modeling ubiquitous physical anatomical surface structures^21,22^, e.g., for the reconstruction of missing anatomical structures or for the design of preformed implants^23,24^. Geometric morphometrics is used in biological sciences to capture and quantify geometric information of biological structures, such as the shape variability of human bones or faces^25,26^. This allowed for the calculation of the scaling factor needed for generating the two sizes of SSM.

**Figure 11:**
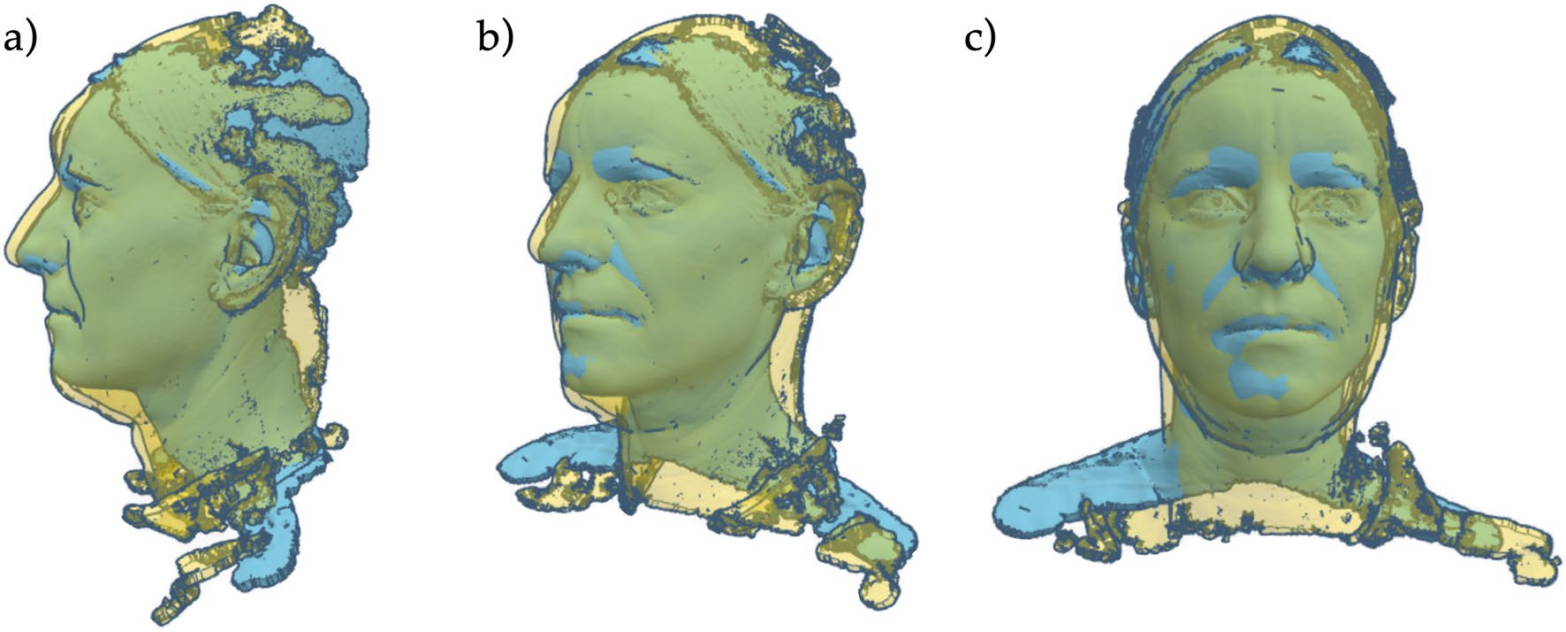
Overlaying two face scans, showing the differences in shape and size from different views: **(a)** the left, **(b)** front left and **(c)** front.

However, the difference between faces of different sizes and shapes (Fig. 11) cannot be completely overcome with one design. Therefore, personal mask designs, with the intention of maximizing seal performance, were developed.

For optimal seal performance, the mask size and design were defined by the position of the menton and glabella of the calculated sex-neutral averaged face. While better seal performance can improve the effectiveness of a mask’s filtering ability, it can also lead to pronounced discomfort by increasing the breathing resistance. This increase in discomfort could lead to less compliance with wearing a mask, as Ferng et al. concluded that it was one of two major factors for low adherence to mask wearing^27^. Therefore, differential pressure to sustain an acceptable level of breathing comfort should be carefully set by application of a suitable filter material with a low follow resistance while still increasing seal performance.

To maximize the possibility for consumers to print mask frames even without the need for skin-compliant filaments, the design was created accordingly. This was archived by only allowing the filter cloth to have contact with the skin as it wraps around the mask frame, held by the horizontal tensioner on the inside and by the retention feet on the outside of the mask. Support structures, which are commonly used to prevent overhangs from deformation by gravity during printing, are not needed with the proposed design. This reduces the amount of wasted material and the postprocessing time, which is commonly not the case for publicly available printable mask designs.

For optimal printing speed, easily printable shapes were used for the retention feet and the tensioner. Only a brim of 8 lines surrounding the first layer of the object was used to optimize bed adhesion and therefore increase printing success. Compared with another 3D-printed mask optimization design^28^ that is meant to enhance wearing comfort, the goal of our design was focused on better seal performance. Bellus3D (Campbell, CA, USA), with their Mask Fitter^29^, followed the same approach. The suggested device is worn on top of a regular mask, which can also be done with the SSM design, although the use of a cut-to-size filter cloth is recommended. Due to the limited supply of medical-grade filter material, other materials could also be used with the PM and SSM designs. It has been shown that household materials are also capable of filtering relevant particle sizes^30^. Konda et al. showed that the filter performance of cotton, natural silk, and chiffon can provide protection, typically above 50% for particle sizes between 10 nm and 6.0 μm, while four-layer silk showed an average efficiency of > 85% for the same particle size range^31^.

A PP copolymer with certified biocompatibility according to USP class VI and ISO 10,993 was used as a material for filament-based 3D printing to meet the highest medical standards^32^. The *Purell* PP used here features high durability, balanced flexibility (tensile modulus 1.0 GPa), allowing the frame to adapt to face contours, and chemical resistance to, for example, common disinfectants. Moreover, its high Vicat softening temperature of 142 °C (VST/A/50, ISO 306) allows it to be steam sterilizable while retaining its shape to enable reuse of the 3D-printed mask frames. Like all polyolefins, PP exhibits unrivaled low raw material and manufacturing costs, making it ideal for global use in medical applications that are accessible to all converters and end-users. The combination of a sustainable solvent-free polymerization process and simple recycling due to its hydrocarbon nature have led to excellent values in life cycle analysis^33^. The certified PP copolymer can be processed with all common FFF printers. Thus, it offers an excellent eco-friendly cost–benefit ratio, both for small decentralized applications and for high-volume production of protective masks or other personal protective equipment.

The leak performance results of the tested masks revealed that the PM showed the lowest leak flow (p < 0.01) of inspired or expired unfiltered air of approximately 10.4 ± 16.4%. While the FFP2 classification implies that for filter cloth, > 94% of passing particles become trapped, we showed in our test that when placed on our face model, 34.9 ± 18.5% of air bypassed the cloth entirely. A comparable leakage was achieved with the SSM (p > 0.68). The SM showed the highest (p < 0.01) leakage, with 84.9 ± 7.7%.

In most studies^16,34^, seal performance tests are conducted by creating aerosol particles, which can then be quantified both in the surrounding air and inside of the mask. The goal of this study was to evaluate seal performance. Therefore, we relied on pressure and flow rate measurements. Furthermore, the use of 3D-printed faces allowed for repeatable results, eliminating inconsistencies by face movements. On the other hand, the lack of compressible skin might have resulted in a loss of seal performance. Nevertheless, from the authors’ point of view, the repeatability outweighs this effect considering that the results are not meant to reflect real-world values but to allow for comparability. The mentioned effects could explain the higher leakage compared to Grinshpun et al., who, however, showed in accordance with our results that the FFP2 mask outperformed the surgical mask^16^. While Cai et al. showed that a personalized mask fitter can improve the contact pressure^28^, to our knowledge, no research has addressed the impact of personalized mask frames based on individual face scans and the resulting seal performance.

The 3D printability of the mask offers three main advantages. First, fast and inexpensive production on demand is possible without a large stock capacity and complexity (e.g., expiring of stored materials), and the risk of uncontrolled material drain (e.g., waste, robbery) can be minimized. The use of filament-based 3D printing is particularly noteworthy, as finished parts can be printed in a one-step production process without the need for posttreatment as required for other additive manufacturing techniques. Second, in-house (quarantined private setting), in-hospital or on-campus production can be easily established if production and supply chains are disrupted, as recognized temporally around the world^35^. Third, the developed PM workflow can be utilized by every company around the world to create personalized masks for their employees at companies with 3D printing facilities. However, the designs cannot yet be recommended for clinical application since mask validation is mandatory according to defined standards (DIN EN 14683:2019-10, DIN EN 149:2009-08, ASTM F2100, NIOSH 42 CFR 84).

**Figure 12:**
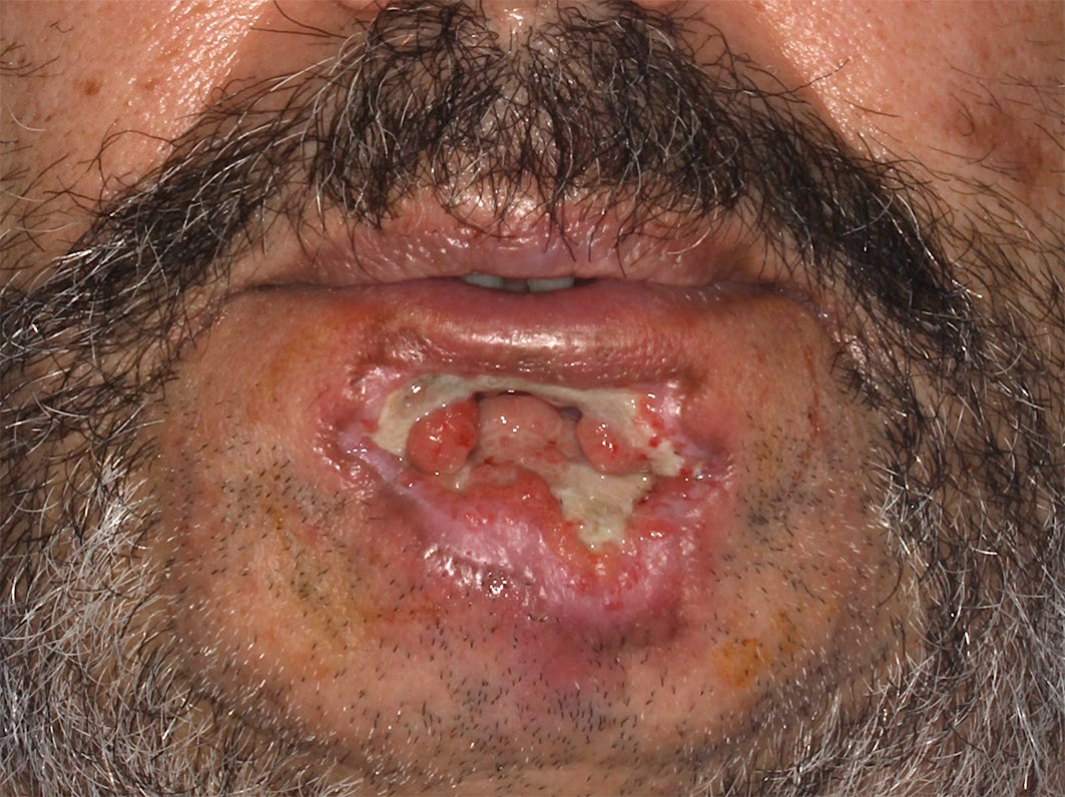
Patient after prolonged COVID-19 treatment requiring respirator use for multiple weeks resulting in severe damage to the skin of the lower lip.

Furthermore, the proposed approach of personalizing medical devices could be adopted to respirators for patients with severe trauma-induced swelling or those who require long-term ventilation. This could lead to improved seal performance and prevent uneven pressure that would result in damage to the patient’s skin (Fig. 12).

Because of the present public health emergency, the material supply for healthcare workers and the general public needs to be realized quickly. 3D printing allows for easy and quick production of PPE, which has already been demonstrated by the wide adoption of additively manufactured face shields^36,37^. To overcome the shortage of face masks, the use of alternative filtering materials and designs for protective face wear is needed^30^. Using 3D scanning software available for smartphones (STL Maker, Scandy LCC, New Orleans, USA) could also allow consumers to follow our workflow and design and print personalized mask frames for their enhanced protection.

## Conclusions

A workflow for designing 3D printable frameworks for an oronasal mask was created, offering a fast and inexpensive on-demand, in-house, or in-hospital production. The seal performance of personalized masks based on individual face scans was superior to that of all tested masks and the FFP2 respirator. Prior to application in a clinical setting, the mask performance has to be approved according to the Emergency Use Authorization (EUA) under section 564 of the Federal Food, Drug, and Cosmetic Act (FD&C Act) and Commission Recommendation (EU) 2020/403 from March 13, 2020.

## Data Availability

The datasets generated and/or analyzed during the current study are available from the corresponding author on reasonable request.
